# Non-steroidal anti-inflammatory drugs induce severe hematologic toxicities in lung cancer patients receiving pemetrexed plus carboplatin: A retrospective cohort study

**DOI:** 10.1371/journal.pone.0171066

**Published:** 2017-02-03

**Authors:** Hitoshi Kawazoe, Akiko Yano, Yuri Ishida, Kenshi Takechi, Hitoshi Katayama, Ryoji Ito, Yoshihiro Yakushijin, Toshihide Moriguchi, Mamoru Tanaka, Akihiro Tanaka, Hiroaki Araki

**Affiliations:** 1 Division of Pharmacy, Ehime University Hospital, Toon, Ehime, Japan; 2 Division of Pharmacy, Komazawa Hospital, Setagaya-ku, Tokyo, Japan; 3 Department of Cardiology, Pulmonology, Hypertension & Nephrology, Ehime University Graduate School of Medicine, Toon, Ehime, Japan; 4 Department of Respiratory Medicine, National Hospital Organization Ehime Medical Center, Toon, Ehime, Japan; 5 Cancer Center, Ehime University Hospital, Toon, Ehime, Japan; University of South Alabama Mitchell Cancer Institute, UNITED STATES

## Abstract

**Purpose:**

As the major toxicity induced by pemetrexed plus carboplatin is severe hematologic toxicities, the aim of this study was to determine the risk factors for severe hematologic toxicities in lung cancer patients.

**Methods:**

We retrospectively investigated data from lung cancer patients who had received pemetrexed plus carboplatin, with or without bevacizumab. This observational study was carried out at Ehime University Hospital using electronic medical records dating from July 2009 to March 2015. Severe hematologic toxicities were defined as grade 3 or 4, according to the Common Terminology Criteria for Adverse Events, version 4.0.

**Results:**

Forty-two patients were included in the study. The incidence of grade 3 or 4 hematologic toxicities during the first cycle of chemotherapy and during all cycles was 19.0% and 16.1%, respectively. Multivariate time-depend generalized estimating equations logistic regression analysis revealed that regular use of non-steroidal anti-inflammatory drugs (NSAIDs) was significantly associated with an increased risk of severe hematologic toxicities during all cycles (adjusted odds ratio (OR): 8.32, 95% confidence interval (CI): 1.27–54.38; *p* = 0.03), whereas creatinine clearance of <45 mL/min was not significantly associated with an increased risk of severe hematologic toxicities during all cycles (adjusted OR: 0.91, 95% CI: 0.25–3.34; *p* = 0.88).

**Conclusions:**

The results suggest that severe hematologic toxicities in patients receiving carboplatin-based pemetrexed may be significantly induced by the inhibition of renal tubular pemetrexed secretion through drug–drug interactions between NSAIDs and pemetrexed rather than through glomerular filtration of pemetrexed, even with moderate to sufficient renal function.

## Introduction

Lung cancer is the most commonly diagnosed type of cancer and the leading cause of cancer-related mortality, both worldwide and in Japan. Pemetrexed, a multitargeted antifolate, is a key drug for patients with non-squamous, non-small cell lung cancer (NSCLC) [1–5]. Pemetrexed in combination with platinum is standard first-line chemotherapy for these patients, and the leading therapy for prolonging survival and improving quality of life compared with third generation chemotherapeutic agents plus platinum. Cisplatin and carboplatin are traditional first and second generation platinum drugs, respectively. Cisplatin is associated with severe non-hematologic and mild hematologic toxicities, including nausea, vomiting, and renal disorder, and should be administered alongside the best available antiemetics [6] and adequate hydration [7]. In contrast, carboplatin produces fewer toxicities than cisplatin, even without the use of supportive therapies to address toxic effects. Carboplatin-based pemetrexed is widely used because of its lower toxicity and short infusion time, making it convenient for outpatient chemotherapy. The pharmacokinetics of pemetrexed depend upon the renal function of patients and concomitant administration of non-steroidal anti-inflammatory drugs (NSAIDs) [8–10]. Several drugs can induce nephrotoxicity, including NSAIDs, zoledronic acid (ZOL), radiocontrast agents, vancomycin, and fibrate-based medicines. On the other hand, angiotensin-converting enzyme inhibitor (ACE) and angiotensin II receptor blocker (ARB) have been shown to have a renoprotective effect [11–15].

The major toxicity induced by pemetrexed plus carboplatin is severe hematologic toxicities, observed in 25.8–40.0% of patients [2, 4]. However, the predictive risk factors for severe hematologic toxicities in patients receiving pemetrexed plus carboplatin remain unclear. Population aging is progressing in Japan and other developed countries, resulting in increased numbers of renally impaired older adults who are taking multiple concomitant oral medications. The safe treatment of this patient population remains a clinically unresolved issue. Identification of the risk factors associated with severe hematologic toxicities in patients receiving pemetrexed plus carboplatin would allow clinicians and pharmacists to better support patients with those risk factors. The aim of this study was therefore to clarify the risk factors for severe hematologic toxicities in cancer patients.

## Methods

### Patients and study design

This retrospective observational study was carried out at Ehime University Hospital using data from electronic medical records dating from July 2009 to March 2015. Patient records were de-identified and analyzed anonymously. We extracted the necessary clinical information on patient demographics, compliance, and hematologic toxicities from patients with NSCLC who had received pemetrexed plus carboplatin, with or without bevacizumab. Pharmacists in hospital and community pharmacies routinely confirmed the compliance of oral medicines including NSAIDs. Pemetrexed was administered at 500 mg/m^2^ by 10 minutes of intravenous infusion every 3 weeks until disease progression, an unacceptable level of toxicities, or patient refusal. The dose of pemetrexed and carboplatin and the treatment schedule were modified at the clinicians’ discretion, according to the toxicity profiles. Carboplatin was concomitantly administered according to the targeted area under the curve (AUC), as estimated by the Calvert formula [16]. The relative dose intensity (RDI) of pemetrexed was defined as the actual dose divided by the planned dose during the period of pemetrexed treatment. Consenting patients with stage IV or relapsed NSCLC were candidates for pemetrexed plus carboplatin therapy. Creatinine clearance (CCr) was calculated using the Cockcroft–Gault formula by adding 0.2 mg/dL to the serum creatinine level measured by the enzymatic peroxidase–antiperoxidase method [17]. Severe hematologic toxicities, leukopenia, neutropenia, anemia, and thrombocytopenia were defined as grade 3 or 4 according to the Common Terminology Criteria for Adverse Events, version 4.0. The nadir of previous hematologic toxicities in each patient was determined as the lowest value recorded during any course of pemetrexed plus carboplatin. In the present study, the frequency of laboratory testing was determined at the clinicians’ discretion.

The study protocol was approved by the ethics committee of Ehime University Hospital (approval number: 1506001) and was conducted in accordance with the Declaration of Helsinki and the Ethical Guidelines for Medical and Health Research involving Human Subjects by the Ministry of Education, Culture, Sports, Science and Technology, and the Ministry of Health, Labour and Welfare of Japan.

### Statistical analysis

Firstly, univariate logistic regression analysis was used to identify risk factors associated with grade 3 or 4 hematologic toxicities per patient. Secondly, the generalized estimating equations (GEE) analysis with time-depend covariates adjustment for the binary multivariate model was used to identify risk factors associated with grade 3 or 4 hematologic toxicities per cycle because analytic number of patient was insufficient for multivariate analysis as well as standard treatment of platinum doublet for NSCLC was from 4 to 6 cycles. The data of working correlation matrix was performed as independent variable. Cases with missing values on the dependent variable, covariates, scale weight variable, or offset variable are always excluded. The receiver operating characteristic (ROC) curve was also used to determine the cut-off value for the continuous variables such as AUC and number of cycles as binary variables. All possible explanatory variables reported in several previous studies were included in the univariate model as independent variables per patient. Patient sex (male or female), age (≥70 or <70 years old), pemetrexed RDI (<75 or ≥75%), co-administered bevacizumab (yes or no), prior chemotherapy (yes or no), treatment line (1st vs 2nd), regular use of NSAIDs (yes or no), ACE/ARBs (yes or no), proton pump inhibitors (PPIs) (yes or no), co-administered ZOL (yes or no), radiocontrast agents (yes or no), baseline hemoglobin (HGB) (<11.6 or ≥11.6 g/dL), and CCr (<45 or ≥45 mL/min) were evaluated as binary variables. This study defined regular use of NSAIDs, ACE/ARBs, and PPIs was defined to take those medicines every day during the study period and also defined co-administered bevacizumab, ZOL, and radiocontrast agents was defined to administer those medicines the day of administration of pemetrexed. The explanatory variables of a confounding factor and/or multicollinearity were excluded in the multivariate model. To identify a confounding factor and multicollinearity, the correlations between explanatory variables and the variance inflation factor values were calculated using Spearman’s correlation coefficient test and the standard least squares method, respectively. Univariate logistic regression analysis and ROC curve was performed using JMP 8.0 (SAS Institute, Tokyo, Japan). The time-depend GEE analysis was performed using IBM SPSS Statistics 21 (IBM Corp., Tokyo, Japan). All *p* values were two-tailed, and *p* < 0.05 was considered significant.

## Results

Forty-two patients and 174 cycles of pemetrexed plus carboplatin were included in the study. Baseline patient characteristics are summarized in Table 1. The median dose of pemetrexed and carboplatin at first cycle was 495 mg/m^2^ [range 457–513 mg/m^2^] and AUC 5 [AUC 4–AUC 6], respectively. Median and range for the number of cycles of carboplatin-based pemetrexed were 4 and 1–8, respectively. The median values for white blood cell, neutrophil, HGB, and platelet counts; aspartate transaminase, alanine transaminase, and CCr at baseline were within the normal reference ranges of our institute.

**Table 1:** Baseline patient characteristics.

Number of patients (n = 42)		
Sex, n (%)	Male	25 (59.5)
	Female	17 (40.5)
Age (years)^a^		67 [27–81]
Body surface area (m^2^)^a^		1.60 [1.21–2.00]
Pemetrexed dose (mg/m^2^)^a^		495 [457–513]
Carboplatin dose (target AUC)^a^		5 [4–6]
Co-administered bevacizumab,	Yes	12 (28.6)
n (%)	No	30 (71.4)
Bevacizumab dose (mg/kg)^a^		15 [13–15]
Prior chemotherapy,	Yes	15 (35.7)
n (%)	No	27 (64.3)
Treatment line^a^		1 [1–2]
Number of cycles^a^		4 [1–8]
Regular use of NSAIDs,	Yes	7 (16.7)
n (%)	No	35 (83.3)
Regular use of ACE/ARBs,	Yes	9 (21.4)
n (%)	No	33 (78.6)
Regular use of PPIs,	Yes	15 (35.7)
n (%)	No	27 (64.3)
Co-administered ZOL,	Yes	5 (11.9)
n (%)	No	37 (88.1)
Co-administered radiocontrast agents,	Yes	16 (38.1)
n (%)	No	26 (61.9)
Baseline WBC (1×10^3^cells/mm^3^)^a^		5.8 [3.2–31.0]
ANC (1×10^3^cells/mm^3^)^a^		3.9 [1.5–28.1]
HGB (g/dL)^a^		12.7 [8.6–15.7]
PLT (1×10^4^cells/mm^3^)^a^		24.8 [12.2–50.8]
Baseline AST (U/L)^a^		23 [11–52]
ALT (U/L)^a^		18 [7–46]
Baseline CCr (mL/min)^a^		60.3 [26.0–91.9]

*AUC* targeted area under the curve estimated by the Calvert formula, *NSAIDs* non-steroidal anti-inflammatory drugs, *ACE* angiotensin-converting enzyme inhibitor, *ARB* angiotensin receptor blocker, *PPIs* proton pump inhibitors, *ZOL* zoledronic acid, *WBC* white blood cell, *ANC* absolute neutrophil count, *HGB* hemoglobin, *PLT* platelet, *AST* aspartate transaminase, *ALT* alanine transaminase, *CCr* creatinine clearance.

^a^Values shown as median [range].

All patients received standard supplementation with oral folic acid (400–500 μg) daily and an intramuscular vitamin B_12_ injection (1,000 μg) every three cycles, according to the package label for pemetrexed. Vancomycin and fibrate-based medicines, which can induce nephrotoxicity, were not administered to patients.

**Fig 1:**
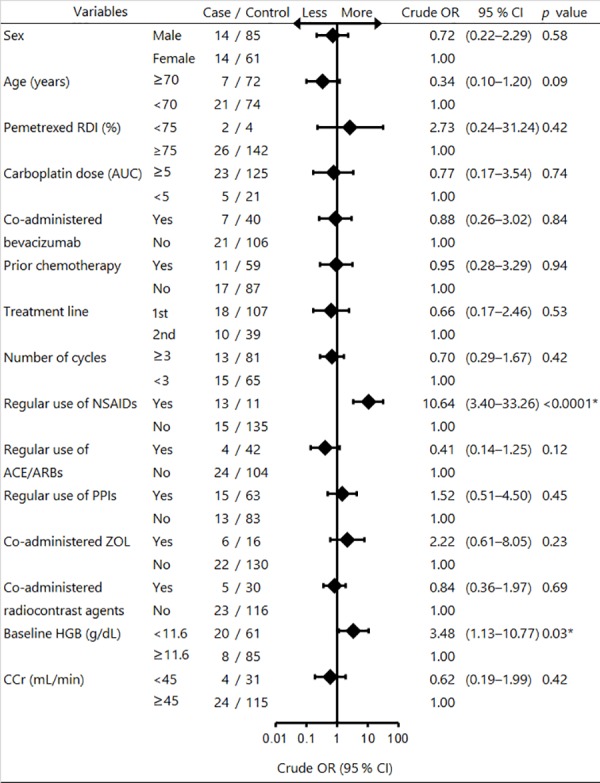
Forest plot of univariate time-depend GEE logistic regression analysis for risk factors associated with severe hematologic toxicities. *OR* odds ratio, *CI* confidence interval, *RDI* relative dose intensity, *AUC* targeted area under the curve estimated by the Calvert formula, *NSAIDs* non-steroidal anti-inflammatory drugs, *ACE* angiotensin-converting enzyme inhibitor, *ARB* angiotensin receptor blocker, *PPIs* proton pump inhibitors, *ZOL* zoledronic acid, *HGB* hemoglobin, *CCr* creatinine clearance. Univariate time-depend GEE logistic regression analysis was used to identify the risk factors associated with severe hematologic toxicities per cycle. *p < 0.05.

**Fig 2:**
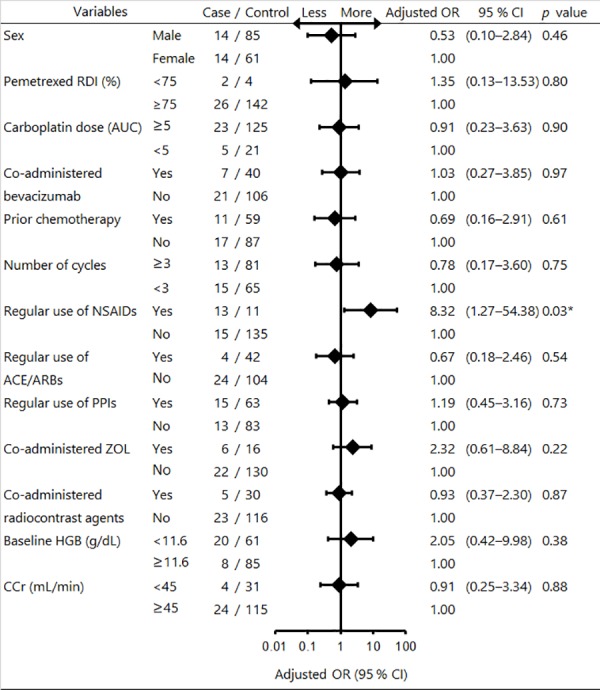
Forest plot of multivariate time-depend GEE logistic regression analysis for risk factors associated with severe hematologic toxicities. Multivariate time-depend GEE logistic regression analysis was used to identify the risk factors associated with severe hematologic toxicities per cycle. *p < 0.05.

The incidence of grade 3 or 4 hematologic toxicities during the first cycle and during all cycles was 19.0% (8/42) and 16.1% (28/174), respectively. The most common incidence of grade 3 or 4 hematologic toxicities during all cycles was neutropenia (10.3%). Univariate logistic regression analysis revealed that there were no risk factors significantly associated with an increased risk of severe hematologic toxicities per patient (data not shown). The optimal cut-off value for AUC and number of cycles was 5 and 3, respectively. The area under the curve of those ROC curve was 0.53 for AUC and 0.57 for cycles, respectively. Spearman’s rank correlation coefficient test revealed the following confounding factors: strong significant positive correlation was observed between prior chemotherapy (yes or no) and treatment line (1st vs 2nd) (0.76; *p* < 0.0001) and between CCr (<45 or ≥45 mL/min) and age (≥70 or <70 years old) (0.52; *p* < 0.0001). We excluded treatment line and age in the multivariate model. The maximum variance inflation factor value between all variables was 3.27, indicating that multicollinearity was not present in this model. The results of univariate and multivariate time-depend GEE analysis to identify risk factors associated with grade 3 or 4 hematologic toxicities per cycle are shown in Figs 1 and 2. The goodness of fit for multivariate time-depend GEE model was the following. Quasi likelihood under independence model criterion (QIC) and corrected quasi likelihood under independence model criterion (QICC) was 156 and 153, respectively. QIC and QICC was computed using the full log quasi-likelihood function. Univariate time-depend GEE logistic regression analysis revealed that regular use of NSAIDs and baseline HGB of <11.6 g/dL were significantly associated with an increased risk of severe hematologic toxicities per cycle (crude odds ratio (OR): 10.64, 95% confidence interval (CI): 3.40–33.26; *p* < 0.0001; and crude OR: 3.48, 95% CI: 1.13–10.77; *p* = 0.03, respectively). Multivariate time-depend GEE logistic regression analysis also revealed that regular use of NSAIDs was significantly associated with an increased risk of severe hematologic toxicities per cycle (adjusted OR: 8.32, 95% CI: 1.27–54.38; *p* = 0.03), whereas CCr of <45 mL/min was not significantly associated with an increased risk of severe hematologic toxicities per cycle (adjusted OR: 0.91, 95% CI: 0.25–3.34; *p* = 0.88).

## Discussion

There is little information available at present on the predictive risk factors for severe hematologic toxicities in patients receiving pemetrexed plus carboplatin in the clinical practice setting. This was a pilot research and the primary objective of this pilot research was to generate the hypotheses for future formal study. The present study showed that the incidence of grade 3 or 4 hematologic toxicities during the first cycle and during all cycles was 19.0% and 16.1%, respectively, figures lower than those observed in previous phase III trials (25.8–40.0%) [2, 4]. We used the time-depend GEE analysis to identify, for the first time, that regular use of NSAIDs was significantly associated with an increased risk of severe hematologic toxicities.

The present study demonstrated that regular use of NSAIDs was significantly associated with an increased risk of severe hematologic toxicities induced by pemetrexed plus carboplatin. To our knowledge, such an association has not previously been described, although similar findings have been reported for methotrexate, which has a similar structure to pemetrexed, an antifolate chemotherapeutic agent that inhibits dihydrofolate reductase [18]. Previous reports showed that co-administration of NSAIDs induced a marked elevation in blood methotrexate concentrations and subsequent severe toxicity [19, 20]. Such effects of NSAIDs might thus induce severe hematologic toxicities through drug–drug interactions between NSAIDs and methotrexate or pemetrexed. Pemetrexed is a hydrophilic anionic compound eliminated unchanged in the urine (70–90%) primarily by active tubular secretion, presumably by human organic anion transporter 3 (hOAT3) [21, 22]. It is suggested that NSAIDs reduce the elimination rate of pemetrexed by inhibiting hOAT3 [21]. Previous studies have also reported that the pharmacokinetics of pemetrexed are modulated by concomitant administration of ibuprofen [10]. However, no evidence to date has shown that co-administration of NSAIDs with pemetrexed has any effect on hematologic toxicities in a clinical setting. According to the package label of pemetrexed, patients with mild to moderate renal insufficiency should avoid taking NSAIDs with short elimination half-lives for a period of 2 days before, the day of, and 2 days following administration of pemetrexed. In the absence of data regarding potential interactions between pemetrexed and NSAIDs with longer half-lives, all patients taking these NSAIDs should interrupt dosing for at least 5 days before, the day of, and 2 days following pemetrexed administration. However, Ikesue et al. [23] reported that co-administration of NSAIDs was not significantly associated with an increased risk of grade 3 or 4 neutropenia in patients receiving pemetrexed plus carboplatin in a univariate analysis. This inconsistency may be the result of several differences in evaluation method of grade 3 or 4 neutropenia or hematologic toxicities, or in the multivariate logistic regression model of different explanatory variables used for analysis. These findings suggest that severe hematologic toxicities in patients receiving carboplatin-based pemetrexed are dependent on regular use of NSAIDs. Taken together, if concomitant administration of an NSAID is necessary for pain control in patients, acetaminophen, an alternative choice of NSAID, should be considered.

We also found that CCr <45 mL/min was not significantly associated with an increased risk of severe hematologic toxicities induced by pemetrexed plus carboplatin. As mentioned above, pharmacokinetic studies have reported that the pharmacokinetics of pemetrexed depend upon the renal function of the patient [8, 9]. CCr values were associated with the clearance and AUC of pemetrexed [9]. Insufficient pemetrexed pharmacokinetic information exists on patients with insufficient renal function, defined as CCr of <45 mL/min. In only 10 patients with malignant pleural mesothelioma with a CCr of 45–60 mL/min, pemetrexed in combination with cisplatin was not tolerable [24]. Similarly, Sakata et al. [25] reported that baseline CCr of <45 mL/min was a risk factor for severe adverse events in 82 Japanese patients with advanced NSCLC receiving pemetrexed monotherapy. This conflicting information may be attributable to several differences in patient groups with or without carboplatin, and to the multivariate logistic regression model of different explanatory variables used for analysis. Moreover, renal tubular secretion of pemetrexed was shown to be about 2.5-fold higher than glomerular filtration in advanced cancer patients with normal renal function [8]. In our study, the incidence of severe hematologic toxicities in all 24 patients who were regularly taking NSAIDs was 54.2% (13/24), even with CCr of ≥45 mL/min. Taken together, severe hematologic toxicities in patients receiving carboplatin-based pemetrexed may be significantly induced by the inhibition of renal tubular secretion of pemetrexed through drug–drug interactions between NSAIDs and pemetrexed via hOAT3 rather than glomerular filtration of pemetrexed, even with moderate to sufficient renal function of CCr ≥45 mL/min.

The present study indicated, for the first time, that regular use of PPIs was not significantly associated with severe hematologic toxicities induced by pemetrexed plus carboplatin. To our knowledge, such a negative association has not previously been described, although similar findings have been reported for methotrexate [26–28]. The proposed mechanisms for drug–drug interactions between methotrexate and PPIs include inhibition of renal H^+^/K^+^-ATPase involved in active tubular secretion of methotrexate or the inhibition of breast cancer resistance protein, which is also involved in mediating methotrexate transport. Such effects of PPIs might thus induce severe hematologic toxicities through drug–drug interactions between pemetrexed and PPIs. This proposed mechanism is controversial, however, even in a high-dose methotrexate setting. Based on these results, the clinical significance of any potential interaction between pemetrexed and PPIs is likely to be limited.

The present study has a slight bias towards carboplatin-induced severe hematologic toxicities. However, carboplatin dose was adjusted by CCr based on the Calvert formula [16]. Calvert et al. [16] reported that AUC of carboplatin dose significantly and positively correlated with carboplatin-induced thrombocytopenia. In our study, the incidence of severe thrombocytopenia was only 3.4% (6/174) during all cycles. Taken together, severe hematologic toxicities in patients receiving carboplatin-based pemetrexed are independent of the effects of carboplatin.

The study had several limitations, the first of which was its retrospective, single-institution study design with a small sample size. The present study revealed that there were no risk factors significantly associated with an increased risk of severe hematologic toxicities per patient. Standard treatment of platinum doublet for NSCLC is from 4 to 6 cycles. So we used the time-depend GEE analysis to adjust the correlation among the same patient data because the hematologic toxicities data was per cycles which included the repeated cycles of chemotherapy. Therefore, the multivariate model is suitable in 13 explanatory variables. Second, we could not fully assess the performance status of patients and the incidence and intensity of non-hematologic toxicities because of the retrospective nature of the study. Such toxicities, including nausea, vomiting, and diarrhea, might be associated with an increased risk of platinum-induced nephrotoxicity. Finally, comorbidities relevant to inherent nephrotoxicity, such as proteinuria, hypocalcemia, and renal tubular acidosis, were not evaluated in the present study, meaning that a degree of bias may have been introduced into our results. Large-scale, multicenter studies are therefore needed to confirm the findings of our study.

In conclusion, this study is the first to clarify the risk factors for severe hematologic toxicities in cancer patients receiving pemetrexed in combination with carboplatin in a clinical setting, using the time-depend GEE analysis. Our findings also suggest that severe hematologic toxicities in patients receiving carboplatin-based pemetrexed may be significantly induced by the inhibition of renal tubular secretion of pemetrexed through drug–drug interactions between NSAIDs and pemetrexed rather than through glomerular filtration of pemetrexed, even with moderate to sufficient renal function.

## Supporting information

S1 File The log files of the statistical analyses from IBM SPSS Statistics 21. (PDF)
