# Supplementary material for: Non-steroidal anti-inflammatory drugs induce severe hematologic toxicities in lung cancer patients receiving pemetrexed plus carboplatin: A retrospective cohort study
Source: PLoS One. 2017 Feb 3;12(2):e0171066. doi: 10.1371/journal.pone.0171066 (PMC5291448; doi:10.1371/journal.pone.0171066)
Supplement: S1 File — (PDF) [file pone.0171066.s001.pdf]

Model Information

Dependent Variable		G3toxicities
Probability Distribution		Binomial
Link Function		Logit
Subject Effect	1	Patient ID
Within-Subject Effect	1	Cycle
Working Correlation Matrix Structure		Independent

Case Processing Summary

	N	Percent
Included	174	100.0%
Excluded	0	0.0%
Total	174	100.0%

Correlated Data Summary

Number of Levels	Subject Effect	Patient ID	42
	Within-Subject	Cycle	8
Number of Subjects			42
Number of Measurements per Subject	Minimum		1
	Maximum		8
Correlation Matrix Dimension			8

Categorical Variable Information

			N	Percent
Dependent Variable	G3toxicities	.0	146	83.9%
		1.0	28	16.1%
		Total	174	100.0%
Factor	Male	.0	75	43.1%
		1.0	99	56.9%
		Total	174	100.0%

Continuous Variable Information

		N	Minimum	Maximum	Mean	Deviation
Covariate	Cycle	174	1.0	8.0	2.868	1.5802

Goodness of Fit

	Value
Quasi Likelihood under Independence Model Criterion (QIC)	161.081
Corrected Quasi Likelihood under Independence Model Criterion	156.892

Tests of Model Effects

	Type III		
Source	Wald Chi-Square	df	Sig.
(Intercept)	30.560	1	.000
Male	.314	1	.576

Parameter Estimates

			95% Wald Confidence Interval		Hypothesis Test				95% Wald Confidence Interval for Exp(B)	
Parameter	B	Std. Error	Lower	Upper	Wald Chi-Square	df	Sig.	Exp(B)	Lower	Upper
(切片)	1.804	.3915	1.036	2.571	21.220	1	.000	6.071	2.819	13.079
[Male=.0]	-.332	.5925	-1.493	.830	.314	1	.576	.718	.225	2.292
[Male=1.0]	0							1		
(Scale)	1									

Model Information

Dependent Variable		G3toxicities
Probability Distribution		Binomial
Link Function		Logit
Subject Effect	1	Patient ID
Within-Subject Effect	1	Cycle
Working Correlation Matrix Structure		Independent

Case Processing Summary

	N	Percent
Included	174	100.0%
Excluded	0	0.0%
Total	174	100.0%

Correlated Data Summary

Number of Levels	Subject Effect	Patient ID	42
	Within-Subject	Cycle	8
Number of Subjects			42
Number of Measurements per Subject	Minimum		1
	Maximum		8
Correlation Matrix Dimension			8

Categorical Variable Information

			N	Percent
Dependent Variable	G3toxicities	.0	146	83.9%
		1.0	28	16.1%
		Total	174	100.0%
Factor	Age70	.0	95	54.6%
		1.0	79	45.4%
		Total	174	100.0%

Continuous Variable Information

		N	Minimum	Maximum	Mean	Deviation
Covariate	Cycle	174	1.0	8.0	2.868	1.5802

Goodness of Fit

	Value
Quasi Likelihood under Independence Model Criterion (QIC)	155.326
Corrected Quasi Likelihood under Independence Model Criterion	151.655

Tests of Model Effects

	Type III		
Source	Wald Chi-Square	df	Sig.
(Intercept)	31.481	1	.000
Age70	2.802	1	.094

Parameter Estimates

			95% Wald Confidence Interval		Hypothesis Test				95% Wald Confidence Interval for Exp(B)	
Parameter	B	Std. Error	Lower	Upper	Wald Chi-Square	df	Sig.	Exp(B)	Lower	Upper

(切片)	2.331	.5356	1.281	3.380	18.938	1	.000	10.286	3.600	29.385
[Age70=.0]	-1.071	.6399	-2.325	.183	2.802	1	.094	.343	.098	1.201
[Age70=1.0]	0							1		
(Scale)	1									

Model Information

Dependent Variable		G3toxicities
Probability Distribution		Binomial
Link Function		Logit
Subject Effect	1	Patient ID
Within-Subject Effect	1	Cycle
Working Correlation Matrix Structure		Independent

Case Processing Summary

	N	Percent
Included	174	100.0%
Excluded	0	0.0%
Total	174	100.0%

Correlated Data Summary

Number of Levels	Subject Effect	Patient ID	42
	Within-Subject	Cycle	8
Number of Subjects			42
Number of Measurements per Subject	Minimum		1
	Maximum		8
Correlation Matrix Dimension			8

Categorical Variable Information

			N	Percent
Dependent Variable	G3toxicities	.0	146	83.9%
		1.0	28	16.1%
		Total	174	100.0%
Factor	RDI75	.0	168	96.6%
		1.0	6	3.4%
		Total	174	100.0%

Continuous Variable Information

		N	Minimum	Maximum	Mean	Deviation
Covariate	Cycle	174	1.0	8.0	2.868	1.5802

Goodness of Fit

	Value
Quasi Likelihood under Independence Model Criterion (QIC)	160.141
Corrected Quasi Likelihood under Independence Model Criterion	156.414

Tests of Model Effects

	Type III		
Source	Wald Chi-Square	df	Sig.
(Intercept)	3.522	1	.061
RDI75	.653	1	.419

Parameter Estimates

			95% Wald Confidence Interval		Hypothesis Test				95% Wald Confidence Interval for Exp(B)	
Parameter	B	Std. Error	Lower	Upper	Wald Chi-Square	df	Sig.	Exp(B)	Lower	Upper
(切片)	.693	1.2247	-1.707	3.094	.320	1	.571	2.000	.181	22.056
[RDI75=.0]	1.005	1.2435	-1.433	3.442	.653	1	.419	2.731	.239	31.244
[RDI75=1.0]	0							1		
(Scale)	1									

Model Information

Dependent Variable		G3toxicities
Probability Distribution		Binomial
Link Function		Logit
Subject Effect	1	Patient ID
Within-Subject Effect	1	Cycle
Working Correlation Matrix Structure		Independent

Case Processing Summary

	N	Percent
Included	174	100.0%
Excluded	0	0.0%
Total	174	100.0%

Correlated Data Summary

Number of Levels	Subject Effect	Patient ID	42
	Within-Subject	Cycle	8
Number of Subjects			42
Number of Measurements per Subject	Minimum		1
	Maximum		8
Correlation Matrix Dimension			8

Categorical Variable Information

			N	Percent
Dependent Variable	G3toxicities	.0	146	83.9%
		1.0	28	16.1%
		Total	174	100.0%
Factor	AUC5	.0	26	14.9%
		1.0	148	85.1%
		Total	174	100.0%

Continuous Variable Information

		N	Minimum	Maximum	Mean	Deviation
Covariate	Cycle	174	1.0	8.0	2.868	1.5802

Goodness of Fit

	Value
Quasi Likelihood under Independence Model Criterion (QIC)	161.424
Corrected Quasi Likelihood under Independence Model Criterion	157.320

Tests of Model Effects

	Type III		
Source	Wald Chi-Square	df	Sig.
(Intercept)	14.087	1	.000
AUC5	.110	1	.740

Parameter Estimates

Parameter	B	Std. Error	95% Wald Confidence Interval		Hypothesis Test			Exp(B)	95% Wald Confidence Interval for Exp(B)	
			Lower	Upper	Wald Chi-Square	df	Sig.		Lower	Upper
(切片)	1.693	.3050	1.095	2.291	30.795	1	.000	5.435	2.989	9.882
[AUC5=.0]	-.258	.7764	-1.780	1.264	.110	1	.740	.773	.169	3.540
[AUC5=1.0]	0							1		
(Scale)	1									

Model Information

Dependent Variable		G3toxicities
Probability Distribution		Binomial
Link Function		Logit
Subject Effect	1	Patient ID
Within-Subject Effect	1	Cycle
Working Correlation Matrix Structure		Independent

Case Processing Summary

	N	Percent
Included	174	100.0%
Excluded	0	0.0%
Total	174	100.0%

Correlated Data Summary

Number of Levels	Subject Effect	Patient ID	42
	Within-Subject	Cycle	8
Number of Subjects			42
Number of Measurements per Subject	Minimum		1
	Maximum		8
Correlation Matrix Dimension			8

Categorical Variable Information

			N	Percent
Dependent Variable	G3toxicities	.0	146	83.9%
		1.0	28	16.1%
		Total	174	100.0%
Factor	Bevacizumab	.0	127	73.0%
		1.0	47	27.0%
		Total	174	100.0%

Continuous Variable Information

		N	Minimum	Maximum	Mean	Deviation
Covariate	Cycle	174	1.0	8.0	2.868	1.5802

Goodness of Fit

	Value
Quasi Likelihood under Independence Model Criterion (QIC)	161.086
Corrected Quasi Likelihood under Independence Model Criterion	157.465

Tests of Model Effects

Source	Type III		
	Wald Chi-Square	df	Sig.
(Intercept)	23.438	1	.000
Bevacizumab	.039	1	.843

Parameter Estimates

Parameter	B	Std. Error	95% Wald Confidence Interval		Hypothesis Test			Exp(B)	95% Wald Confidence Interval for Exp(B)	
			Lower	Upper	Wald Chi-Square	df	Sig.		Lower	Upper
(切片)	1.743	.5774	.611	2.875	9.111	1	.003	5.714	1.843	17.721
[Bevacizumab=.0]	-.124	.6267	-1.352	1.104	.039	1	.843	.883	.259	3.017
[Bevacizumab=1.0]	0							1		
(Scale)	1									

Model Information

Dependent Variable		G3toxicities
Probability Distribution		Binomial
Link Function		Logit
Subject Effect	1	Patient ID
Within-Subject Effect	1	Cycle
Working Correlation Matrix Structure		Independent

Case Processing Summary

	N	Percent
Included	174	100.0%
Excluded	0	0.0%
Total	174	100.0%

Correlated Data Summary

Number of Levels	Subject Effect	Patient ID	42
	Within-Subject	Cycle	8
Number of Subjects			42
Number of Measurements per Subject	Minimum		1
	Maximum		8
Correlation Matrix Dimension			8

Categorical Variable Information

			N	Percent
Dependent Variable	G3toxicities	.0	146	83.9%
		1.0	28	16.1%
		Total	174	100.0%
Factor	Priorchemotherapy	.0	104	59.8%
		1.0	70	40.2%
		Total	174	100.0%

Continuous Variable Information

		N	Minimum	Maximum	Mean	Deviation
Covariate	Cycle	174	1.0	8.0	2.868	1.5802

Goodness of Fit

	Value
Quasi Likelihood under Independence Model Criterion (QIC)	162.114
Corrected Quasi Likelihood under Independence Model Criterion	157.522

Tests of Model Effects

	Type III

Source	Wald Chi-Square	df	Sig.
(Intercept)	27.083	1	.000
Priorchemotherapy	.006	1	.941

Parameter Estimates

Parameter	B	Std. Error	95% Wald Confidence Interval		Hypothesis Test			Exp(B)	95% Wald Confidence Interval for Exp(B)	
			Lower	Upper	Wald Chi-Square	df	Sig.		Lower	Upper
(切片)	1.680	.5367	.628	2.731	9.795	1	.002	5.364	1.873	15.356
[Priorchemotherapy=.0]	-.047	.6321	-1.286	1.192	.006	1	.941	.954	.276	3.293
[Priorchemotherapy=1.0]	0							1		
(Scale)	1									

Model Information

Dependent Variable		G3toxicities
Probability Distribution		Binomial
Link Function		Logit
Subject Effect	1	Patient ID
Within-Subject Effect	1	Cycle
Working Correlation Matrix Structure		Independent

Case Processing Summary

	N	Percent
Included	174	100.0%
Excluded	0	0.0%
Total	174	100.0%

Correlated Data Summary

Number of Levels	Subject Effect	Patient ID	42
	Within-Subject	Cycle	8
Number of Subjects			42
Number of Measurements per Subject	Minimum		1
	Maximum		8
Correlation Matrix Dimension			8

Categorical Variable Information

			N	Percent
Dependent Variable	G3toxicities	.0	146	83.9%
		1.0	28	16.1%
		Total	174	100.0%
Factor	@1stline	.0	49	28.2%
		1.0	125	71.8%
		Total	174	100.0%

Continuous Variable Information

		N	Minimum	Maximum	Mean	Deviation
Covariate	Cycle	174	1.0	8.0	2.868	1.5802

Goodness of Fit

	Value
Quasi Likelihood under Independence Model Criterion (QIC)	161.528
Corrected Quasi Likelihood under Independence Model Criterion	156.629

Tests of Model Effects

Source	Type III		
	Wald Chi-Square	df	Sig.
(Intercept)	21.687	1	.000
@1stline	.390	1	.532

Parameter Estimates

Parameter	B	Std. Error	95% Wald Confidence Interval		Hypothesis Test			Exp(B)	95% Wald Confidence Interval for Exp(B)	
			Lower	Upper	Wald Chi-Square	df	Sig.		Lower	Upper
(切片)	1.782	.3325	1.131	2.434	28.740	1	.000	5.944	3.098	11.406
[@1stline=.0]	-.421	.6750	-1.744	.902	.390	1	.532	.656	.175	2.463
[@1stline=1.0]	0							1		
(Scale)	1									

Model Information

Dependent Variable		G3toxicities
Probability Distribution		Binomial
Link Function		Logit
Subject Effect	1	Patient ID
Within-Subject Effect	1	Cycle
Working Correlation Matrix Structure		Independent

Case Processing Summary

	N	Percent
Included	174	100.0%
Excluded	0	0.0%
Total	174	100.0%

Correlated Data Summary

Number of Levels	Subject Effect	Patient ID	42
	Within-Subject	Cycle	8
Number of Subjects			42
Number of Measurements per Subject	Minimum		1
	Maximum		8
Correlation Matrix Dimension			8

Categorical Variable Information

			N	Percent
Dependent Variable	G3toxicities	.0	146	83.9%
		1.0	28	16.1%
		Total	174	100.0%
Factor	Cycle3	.0	80	46.0%
		1.0	94	54.0%
		Total	174	100.0%

Continuous Variable Information

		N	Minimum	Maximum	Mean	Deviation
Covariate	Cycle	174	1.0	8.0	2.868	1.5802

Goodness of Fit

	Value

Quasi Likelihood under Independence Model Criterion (QIC)	159.175
Corrected Quasi Likelihood under Independence Model Criterion	156.762

Tests of Model Effects

Source	Type III Wald Chi-Square	df	Sig.
(Intercept)	30.954	1	.000
Cycle3	.657	1	.418

Parameter Estimates

Parameter	B	Std. Error	95% Wald Confidence Interval Lower	95% Wald Confidence Interval Upper	Hypothesis Test Wald Chi-Square	Hypothesis Test df	Hypothesis Test Sig.	Exp(B)	95% Wald Confidence Interval for Exp(B) Lower	95% Wald Confidence Interval for Exp(B) Upper
(切片)	1.829	.3966	1.052	2.607	21.284	1	.000	6.231	2.864	13.555
[Cycle3=.0]	-.363	.4480	-1.241	.515	.657	1	.418	.695	.289	1.673
[Cycle3=1.0]	0							1		
(Scale)	1									

Model Information

Dependent Variable		G3toxicities
Probability Distribution		Binomial
Link Function		Logit
Subject Effect	1	Patient ID
Within-Subject Effect	1	Cycle
Working Correlation Matrix Structure		Independent

Case Processing Summary

	N	Percent
Included	174	100.0%
Excluded	0	0.0%
Total	174	100.0%

Correlated Data Summary

Number of Levels	Subject Effect	Patient ID	42
	Within-Subject	Cycle	8
Number of Subjects			42
Number of Measurements per Subject	Minimum		1
	Maximum		8
Correlation Matrix Dimension			8

Categorical Variable Information

			N	Percent
Dependent Variable	G3toxicities	.0	146	83.9%
		1.0	28	16.1%
		Total	174	100.0%
Factor	NSAIDs	.0	150	86.2%
		1.0	24	13.8%
		Total	174	100.0%

Continuous Variable Information

		N	Minimum	Maximum	Mean	Deviation
Covariate	Cycle	174	1.0	8.0	2.868	1.5802

Goodness of Fit

	Value
Quasi Likelihood under Independence Model Criterion (QIC)	136.687
Corrected Quasi Likelihood under Independence Model Criterion	134.629

Tests of Model Effects

Source	Type III Wald Chi-Square	df	Sig.
(Intercept)	10.637	1	.001
NSAIDs	16.520	1	.000

Parameter Estimates

Parameter	B	Std. Error	95% Wald Confidence Interval Lower	95% Wald Confidence Interval Upper	Hypothesis Test Wald Chi-Square	Hypothesis Test df	Hypothesis Test Sig.	Exp(B)	95% Wald Confidence Interval for Exp(B) Lower	95% Wald Confidence Interval for Exp(B) Upper
(切片)	-.167	.4980	-1.143	.809	.113	1	.737	.846	.319	2.246
[NSAIDs=.0]	2.364	.5817	1.224	3.504	16.520	1	.000	10.636	3.401	33.261
[NSAIDs=1.0]	0							1		
(Scale)	1									

Model Information

Dependent Variable		G3toxicities
Probability Distribution		Binomial
Link Function		Logit
Subject Effect	1	Patient ID
Within-Subject Effect	1	Cycle
Working Correlation Matrix Structure		Independent

Case Processing Summary

	N	Percent
Included	174	100.0%
Excluded	0	0.0%
Total	174	100.0%

Correlated Data Summary

Number of Levels	Subject Effect	Patient ID	42
	Within-Subject	Cycle	8
Number of Subjects			42
Number of Measurements per Subject	Minimum		1
	Maximum		8
Correlation Matrix Dimension			8

Categorical Variable Information

			N	Percent
Dependent Variable	G3toxicities	.0	146	83.9%
		1.0	28	16.1%
		Total	174	100.0%
Factor	ACEARB	.0	128	73.6%
		1.0	46	26.4%
		Total	174	100.0%

Continuous Variable Information

		N	Minimum	Maximum	Mean	Deviation
Covariate	Cycle	174	1.0	8.0	2.868	1.5802

Goodness of Fit

	Value
Quasi Likelihood under Independence Model Criterion (QIC)	156.751
Corrected Quasi Likelihood under Independence Model Criterion	154.720

Tests of Model Effects

Source	Type III		
	Wald Chi-Square	df	Sig.
(Intercept)	43.512	1	.000
ACEARB	2.456	1	.117

Parameter Estimates

Parameter	B	Std. Error	95% Wald Confidence Interval		Hypothesis Test			Exp(B)	95% Wald Confidence Interval for Exp(B)	
			Lower	Upper	Wald Chi-Square	df	Sig.		Lower	Upper
(切片)	2.351	.4605	1.449	3.254	26.078	1	.000	10.500	4.258	25.890
[ACEARB=.0]	-.885	.5647	-1.992	.222	2.456	1	.117	.413	.136	1.248
[ACEARB=1.0]	0							1		
(Scale)	1									

Model Information

Dependent Variable		G3toxicities
Probability Distribution		Binomial
Link Function		Logit
Subject Effect	1	Patient ID
Within-Subject Effect	1	Cycle
Working Correlation Matrix Structure		Independent

Case Processing Summary

	N	Percent
Included	174	100.0%
Excluded	0	0.0%
Total	174	100.0%

Correlated Data Summary

Number of Levels	Subject Effect	Patient ID	42
	Within-Subject	Cycle	8
Number of Subjects			42
Number of Measurements per Subject	Minimum		1
	Maximum		8
Correlation Matrix Dimension			8

Categorical Variable Information

			N	Percent
Dependent Variable	G3toxicities	.0	146	83.9%
		1.0	28	16.1%
		Total	174	100.0%
Factor	PPI	.0	96	55.2%
		1.0	78	44.8%
		Total	174	100.0%

Continuous Variable Information

		N	Minimum	Maximum	Mean	Deviation
Covariate	Cycle	174	1.0	8.0	2.868	1.5802

Goodness of Fit

	Value
Quasi Likelihood under Independence Model Criterion (QIC)	160.218
Corrected Quasi Likelihood under Independence Model Criterion	156.509

Tests of Model Effects

Source	Type III		
	Wald Chi-Square	df	Sig.
(Intercept)	31.019	1	.000
PPI	.572	1	.449

Parameter Estimates

Parameter	B	Std. Error	95% Wald Confidence Interval		Hypothesis Test			Exp(B)	95% Wald Confidence Interval for Exp(B)	
			Lower	Upper	Wald Chi-Square	df	Sig.		Lower	Upper
(切片)	1.435	.4440	.565	2.305	10.446	1	.001	4.200	1.759	10.028
[PPI=.0]	.419	.5536	-.666	1.504	.572	1	.449	1.520	.514	4.499
[PPI=1.0]	0							1		
(Scale)	1									

Model Information

Dependent Variable		G3toxicities
Probability Distribution		Binomial
Link Function		Logit
Subject Effect	1	Patient ID
Within-Subject Effect	1	Cycle
Working Correlation Matrix Structure		Independent

Case Processing Summary

	N	Percent
Included	174	100.0%
Excluded	0	0.0%
Total	174	100.0%

Correlated Data Summary

Number of Levels	Subject Effect	Patient ID	42
	Within-Subject	Cycle	8
Number of Subjects			42
Number of Measurements per Subject	Minimum		1
	Maximum		8
Correlation Matrix Dimension			8

Categorical Variable Information

			N	Percent
Dependent Variable	G3toxicities	.0	146	83.9%
		1.0	28	16.1%
		Total	174	100.0%

Factor	ZOL	.0	152	87.4%
		1.0	22	12.6%
		Total	174	100.0%

Continuous Variable Information

		N	Minimum	Maximum	Mean	Deviation
Covariate	Cycle	174	1.0	8.0	2.868	1.5802

Goodness of Fit

	Value
Quasi Likelihood under Independence Model Criterion (QIC)	158.624
Corrected Quasi Likelihood under Independence Model Criterion	155.477

Tests of Model Effects

	Type III		
Source	Wald Chi-Square	df	Sig.
(Intercept)	11.606	1	.001
ZOL	1.463	1	.227

Parameter Estimates

			95% Wald Confidence Interval		Hypothesis Test				95% Wald Confidence Interval for Exp(B)	
Parameter	B	Std. Error	Lower	Upper	Wald Chi-Square	df	Sig.	Exp(B)	Lower	Upper
(切片)	.981	.6789	-.350	2.311	2.087	1	.149	2.667	.705	10.090
[ZOL=.0]	.796	.6579	-.494	2.085	1.463	1	.227	2.216	.610	8.046
[ZOL=1.0]	0							1		
(Scale)	1									

Model Information

Dependent Variable		G3toxicities
Probability Distribution		Binomial
Link Function		Logit
Subject Effect	1	Patient ID
Within-Subject Effect	1	Cycle
Working Correlation Matrix Structure		Independent

Case Processing Summary

	N	Percent
Included	174	100.0%
Excluded	0	0.0%
Total	174	100.0%

Correlated Data Summary

Number of Levels	Subject Effect	Patient ID	42
	Within-Subject	Cycle	8
Number of Subjects			42
Number of Measurements per Subject	Minimum		1
	Maximum		8
Correlation Matrix Dimension			8

Categorical Variable Information

			N	Percent
Dependent Variable	G3toxicities	.0	146	83.9%
		1.0	28	16.1%
		Total	174	100.0%
Factor	Radiocontrast	.0	139	79.9%
		1.0	35	20.1%
		Total	174	100.0%

Continuous Variable Information

		N	Minimum	Maximum	Mean	Deviation
Covariate	Cycle	174	1.0	8.0	2.868	1.5802

Goodness of Fit

	Value
Quasi Likelihood under Independence Model Criterion (QIC)	158.881
Corrected Quasi Likelihood under Independence Model Criterion	157.426

Tests of Model Effects

	Type III		
Source	Wald Chi-Square	df	Sig.
(Intercept)	27.488	1	.000
Radiocontrast	.159	1	.690

Parameter Estimates

			95% Wald Confidence Interval		Hypothesis Test				95% Wald Confidence Interval for Exp(B)	
Parameter	B	Std. Error	Lower	Upper	Wald Chi-Square	df	Sig.	Exp(B)	Lower	Upper
(切片)	1.792	.4595	.891	2.692	15.207	1	.000	6.000	2.438	14.766
[Radiocontrast=.0]	-.174	.4349	-1.026	.679	.159	1	.690	.841	.358	1.971
[Radiocontrast=1.0]	0							1		
(Scale)	1									

Model Information

Dependent Variable		G3toxicities
Probability Distribution		Binomial
Link Function		Logit
Subject Effect	1	Patient ID
Within-Subject Effect	1	Cycle
Working Correlation Matrix Structure		Independent

Case Processing Summary

	N	Percent
Included	174	100.0%
Excluded	0	0.0%
Total	174	100.0%

Correlated Data Summary

Number of Levels	Subject Effect	Patient ID	42
	Within-Subject	Cycle	8
Number of Subjects			42
Number of Measurements per Subject	Minimum		1
	Maximum		8
Correlation Matrix Dimension			8

Categorical Variable Information

			N	Percent
Dependent Variable	G3toxicities	.0	146	83.9%
		1.0	28	16.1%
		Total	174	100.0%
Factor	HGB116	.0	93	53.4%
		1.0	81	46.6%
		Total	174	100.0%

Continuous Variable Information

		N	Minimum	Maximum	Mean	Deviation
Covariate	Cycle	174	1.0	8.0	2.868	1.5802

Goodness of Fit

	Value
Quasi Likelihood under Independence Model Criterion (QIC)	152.239
Corrected Quasi Likelihood under Independence Model Criterion	149.087

Tests of Model Effects

	Type III		
Source	Wald Chi-Square	df	Sig.
(Intercept)	34.073	1	.000
HGB116	4.696	1	.030

Parameter Estimates

			95% Wald Confidence Interval		Hypothesis Test				95% Wald Confidence Interval for Exp(B)	
Parameter	B	Std. Error	Lower	Upper	Wald Chi-Square	df	Sig.	Exp(B)	Lower	Upper
(切片)	1.115	.3708	.388	1.842	9.046	1	.003	3.050	1.475	6.308
[HGB116=.0]	1.248	.5759	.119	2.377	4.696	1	.030	3.484	1.127	10.771
[HGB116=1.0]	0							1		
(Scale)	1									

Model Information

Dependent Variable		G3toxicities
Probability Distribution		Binomial
Link Function		Logit
Subject Effect	1	Patient ID
Within-Subject Effect	1	Cycle
Working Correlation Matrix Structure		Independent

Case Processing Summary

	N	Percent
Included	174	100.0%
Excluded	0	0.0%
Total	174	100.0%

Correlated Data Summary

Number of Levels	Subject Effect	Patient ID	42
	Within-Subject	Cycle	8
Number of Subjects			42
Number of Measurements per Subject	Minimum		1
	Maximum		8
Correlation Matrix Dimension			8

Categorical Variable Information

			N	Percent
Dependent Variable	G3toxicities	.0	146	83.9%
		1.0	28	16.1%
		Total	174	100.0%
Factor	CCr45	.0	139	79.9%
		1.0	35	20.1%
		Total	174	100.0%

Continuous Variable Information

		N	Minimum	Maximum	Mean	Deviation
Covariate	Cycle	174	1.0	8.0	2.868	1.5802

Goodness of Fit

	Value
Quasi Likelihood under Independence Model Criterion (QIC)	159.001
Corrected Quasi Likelihood under Independence Model Criterion	156.780

Tests of Model Effects

	Type III		
Source	Wald Chi-Square	df	Sig.
(Intercept)	35.726	1	.000
CCr45	.648	1	.421

Parameter Estimates

			95% Wald Confidence Interval		Hypothesis Test				95% Wald Confidence Interval for Exp(B)	
Parameter	B	Std. Error	Lower	Upper	Wald Chi-Square	df	Sig.	Exp(B)	Lower	Upper
(切片)	2.048	.4990	1.070	3.026	16.842	1	.000	7.750	2.915	20.607
[CCr45=.0]	-.481	.5973	-1.651	.690	.648	1	.421	.618	.192	1.993
[CCr45=1.0]	0							1		
(Scale)	1									

Model Information

Dependent Variable		G3toxicities
Probability Distribution		Binomial
Link Function		Logit
Subject Effect	1	Patient ID
Within-Subject Effect	1	Cycle
	2	RDI75
	3	AUC5
	4	Bevacizumab
	5	Priorchemothera
	6	Cycle3
	7	NSAIDs
	8	ACEARB
	9	PPI
	10	ZOL
	11	Radiocontrast
	12	HGB116

	13	CCr45
Working Correlation Matrix Structure		Independent

Case Processing Summary

	N	Percent
Included	174	100.0%
Excluded	0	0.0%
Total	174	100.0%

Correlated Data Summary

Number of Levels	Subject Effect	Patient ID	42
	Within-Subject Effect	Cycle	8
		RDI75	2
		AUC5	2
		Bevacizumab	2
		Priorchemothera	2
		Cycle3	2
		NSAIDs	2
		ACEARB	2
		PPI	2
		ZOL	2
		Radiocontrast	2
		HGB116	2
		CCr45	2
Number of Subjects			42
Number of Measurements per Subject	Minimum		1
	Maximum		8
Correlation Matrix Dimension			151

Categorical Variable Information

			N	Percent
Dependent Variable	G3toxicities	.0	146	83.9%
		1.0	28	16.1%
		Total	174	100.0%
Factor	CCr45	.0	139	79.9%
		1.0	35	20.1%
		Total	174	100.0%
	Male	.0	75	43.1%
		1.0	99	56.9%
		Total	174	100.0%
	Age70	.0	95	54.6%
		1.0	79	45.4%
		Total	174	100.0%
	RDI75	.0	168	96.6%
		1.0	6	3.4%
		Total	174	100.0%
	AUC5	.0	26	14.9%
		1.0	148	85.1%
		Total	174	100.0%
	Bevacizumab	.0	127	73.0%
		1.0	47	27.0%
		Total	174	100.0%
	Priorchemotherapy	.0	104	59.8%
		1.0	70	40.2%
		Total	174	100.0%
	@1stline	.0	49	28.2%
		1.0	125	71.8%
		Total	174	100.0%
	Cycle3	.0	80	46.0%
		1.0	94	54.0%
		Total	174	100.0%
	NSAIDs	.0	150	86.2%
		1.0	24	13.8%
		Total	174	100.0%
	ACEARB	.0	128	73.6%
		1.0	46	26.4%
		Total	174	100.0%
	PPI	.0	96	55.2%
		1.0	78	44.8%
		Total	174	100.0%
	ZOL	.0	152	87.4%
		1.0	22	12.6%
		Total	174	100.0%
	Radiocontrast	.0	139	79.9%
		1.0	35	20.1%
		Total	174	100.0%
	HGB116	.0	93	53.4%
		1.0	81	46.6%
		Total	174	100.0%

Continuous Variable Information

		N	Minimum	Maximum	Mean	Deviation
Covariate	Cycle	174	1.0	8.0	2.868	1.5802

Goodness of Fit

	Value
Quasi Likelihood under Independence Model Criterion (QIC)	156.338
Corrected Quasi Likelihood under Independence Model Criterion	153.273

Tests of Model Effects

	Type III		
Source	Wald Chi-Square	df	Sig.
(Intercept)	.635	1	.426
Male	.552	1	.457
RDI75	.066	1	.798
AUC5	.018	1	.895
Bevacizumab	.002	1	.969
Priorchemotherapy	.258	1	.612
Cycle3	.099	1	.753
NSAIDs	4.891	1	.027
ACEARB	.369	1	.544
PPI	.120	1	.729
ZOL	1.524	1	.217
Radiocontrast	.027	1	.869
HGB116	.784	1	.376
CCr45	.021	1	.884
Cycle	.009	1	.925

Parameter Estimates

			95% Wald Confidence Interval		Hypothesis Test				95% Wald Confidence Interval for Exp(B)	
Parameter	B	Std. Error	Lower	Upper	Wald Chi-Square	df	Sig.	Exp(B)	Lower	Upper
(切片)	-.377	2.1026	-4.499	3.744	.032	1	.858	.686	.011	42.248
[Male=.0]	-.638	.8589	-2.322	1.045	.552	1	.457	.528	.098	2.844
[Male=1.0]	0							1		
[RDI75=.0]	.301	1.1756	-2.003	2.605	.066	1	.798	1.351	.135	13.534
[RDI75=1.0]	0							1		
[AUC5=.0]	-.093	.7055	-1.476	1.289	.018	1	.895	.911	.229	3.630
[AUC5=1.0]	0							1		
[Bevacizumab=.0]	.026	.6740	-1.295	1.347	.002	1	.969	1.027	.274	3.847
[Bevacizumab=1.0]	0							1		
[Priorchemotherapy=.0]	-.373	.7353	-1.814	1.068	.258	1	.612	.689	.163	2.909
[Priorchemotherapy=1.0]	0							1		
[Cycle3=.0]	-.244	.7785	-1.770	1.281	.099	1	.753	.783	.170	3.602
[Cycle3=1.0]	0							1		
[NSAIDs=.0]	2.118	.9579	.241	3.996	4.891	1	.027	8.319	1.273	54.378
[NSAIDs=1.0]	0							1		
[ACEARB=.0]	-.404	.6653	-1.708	.900	.369	1	.544	.668	.181	2.459
[ACEARB=1.0]	0							1		
[PPI=.0]	.173	.4985	-.804	1.150	.120	1	.729	1.189	.447	3.158
[PPI=1.0]	0							1		
[ZOL=.0]	.842	.6822	-.495	2.179	1.524	1	.217	2.322	.610	8.841
[ZOL=1.0]	0							1		
[Radiocontrast=.0]	-.077	.4648	-.988	.834	.027	1	.869	.926	.372	2.303
[Radiocontrast=1.0]	0							1		
[HGB116=.0]	.716	.8086	-.869	2.301	.784	1	.376	2.047	.419	9.985
[HGB116=1.0]	0							1		
[CCr45=.0]	-.097	.6643	-1.399	1.205	.021	1	.884	.908	.247	3.337
[CCr45=1.0]	0							1		
Cycle	.031	.3327	-.621	.683	.009	1	.925	1.032	.538	1.981
(Scale)	1									
